# Improving Access to Take-Home Naloxone for Service Users at Risk of Opioid Overdose in an Adult Home Treatment Team: A Quality Improvement Project

**DOI:** 10.1192/bjo.2026.11415

**Published:** 2026-06-30

**Authors:** Tze Lau, Mario Lepore, Elsie Jonas-Nartey, Yuet Ting To, John Bainton

**Affiliations:** South London and Maudsley NHS Foundation Trust, London, United Kingdom

## Abstract

**Aims:**

Drug-related deaths in England and Wales continue to rise, with opioids remaining the most frequently implicated substances. Individuals receiving care from mental health services commonly experience co-occurring substance use disorders, with overdose risk heightened by increasing contamination of street drugs with potent synthetic opioids.

15% of service users under the care of Home Treatment Teams (HTTs) experience substance misuse, making this an important setting for harm-reduction interventions.

Take-home naloxone (THN), an opioid receptor antagonist used in emergency treatment of opioid overdose, is an evidence-based harm-reduction intervention. Patient Group Directions (PGDs) are legal frameworks that allow authorised non-medical healthcare professionals tosupply or administer specified medicines. Although PGDs have facilitated widespread THN provision in addiction services, implementation within HTT settings remains limited.

This quality improvement project aimed to increase access to THN for eligible service users within an adult HTT.

**Methods:**

Electronic records for 30 consecutive service users receiving care from the HTT were reviewed to assess substance use patterns, eligibility for THN and whether THN was offered.

A PGD for THN supply was introduced in collaboration with pharmacy services. Readily accessible THN stock was established within the team’s medication cupboard to facilitate prompt provision. Multidisciplinary teaching sessions were delivered to staff, focusing on overdose risk, THN eligibility criteria and service user education.

Following implementation, electronic records for a further 30 consecutive service users were reviewed using the same criteria, and outcomes compared with baseline data.

**Results:**

At baseline, 13.3% of service users (n=4) met eligibility for THN, of whom 50% (n=2) were offered THN. In both cases, THN was supplied following medical review.

Following the intervention, 16.7% of service users (n=5) were eligible for THN, with 80% (n=4) being offered THN by a combination of medical and nursing professionals.

**Conclusion:**

A clinically significant proportion of HTT service users were identified as being at risk of opioid overdose. The introduction of focused harm-reduction interventions, including a PGD, improved access to THN, and targeted staff education, was associated with increased provision of THN within the HTT setting. While THN is commonly used within addiction services, we are the first team providing care for general adults in our trust to implement this as part of routine care.

Further work is required to optimise service user uptake, address barriers including stigma and limited overdose awareness, and implement interventions across wider community mental health services. For interested teams, we recommend liaising with pharmacy services to explore local PGDs.

